# Supplementary material for: Systematic functional interrogation of SARS-CoV-2 host factors using Perturb-seq
Source: Nat Commun. 2023 Oct 6;14:6245. doi: 10.1038/s41467-023-41788-4 (PMC10558542; doi:10.1038/s41467-023-41788-4)
Supplement: Supplementary file 1 — Supplementary Information File [file 41467_2023_41788_MOESM1_ESM.pdf]

Systematic functional interrogation of SARS-CoV-2 host factors using Perturb-seq

Sara Sunshine[1], Andreas S. Puschnik[2], Joseph M. Replogle[3,4], Matthew T. Laurie[1], Jamin Liu[1,5], Beth Shoshana Zha[6], James K. Nuñez[3,7], Janie R. Byrum[2], Aidan H. McMorrow[2], Matthew B. Frieman[8], Juliane Winkler[9,10], Xiaojie Qiu[4,11], Oren S. Rosenberg[12], Manuel D. Leonetti[2], Chun Jimmie Ye[13,14,15,16,17], Jonathan S. Weissman[4,11,18,19], Joseph L. DeRisi[1,2], and Marco Y. Hein[2,3,20,21,22]

1 Department of Biochemistry and Biophysics, University of California San Francisco, San Francisco, CA, USA
2 Chan Zuckerberg Biohub, San Francisco, San Francisco, CA, USA
3 Department of Cellular and Molecular Pharmacology, University of California, San Francisco, San Francisco, CA, USA
4 Whitehead Institute for Biomedical Research, Cambridge, MA, USA
5 University of California, Berkeley-UCSF Joint Graduate Program in Bioengineering, San Francisco, CA, USA
6 Department of Medicine, Division of Pulmonary, Critical Care, Allergy and Sleep Medicine, University of California, San Francisco, San Francisco, CA, USA
7 Department of Molecular & Cell Biology, University of California, Berkeley, Berkeley, CA, USA
8 Department of Microbiology and Immunology, Center for Pathogen Research, University of Maryland School of Medicine, Baltimore, MD, USA
9 Department of Cell and Tissue Biology, University of California, San Francisco, San Francisco, CA, USA
10 Center for Cancer Research, Medical University of Vienna, Vienna, Austria
11 Howard Hughes Medical Institute, Massachusetts Institute of Technology, Cambridge, MA, USA
12 Department of Microbiology and Immunology, University of California, San Francisco, San Francisco, CA, USA
13 Division of Rheumatology, Department of Medicine, University of California, San Francisco, San Francisco, CA, USA
14 Institute of Human Genetics, University of California San Francisco, San Francisco, CA, USA
15 Department of Epidemiology and Biostatistics, University of California, San Francisco, San Francisco, CA, USA
16 Bakar Computational Health Sciences Institute, University of California, San Francisco, San Francisco, CA, USA
17 Parker Institute for Cancer Immunotherapy, San Francisco, CA, USA
18 Department of Biology, Massachusetts Institute of Technology, Cambridge, MA, USA
19 David H. Koch Institute for Integrative Cancer Research, Massachusetts Institute of Technology, Cambridge, MA, USA
20 Howard Hughes Medical Institute, University of California, San Francisco, San Francisco, CA, USA
21 Max Perutz Labs, Vienna Biocenter Campus (VBC), Vienna, Austria
22 Medical University of Vienna, Center for Medical Biochemistry, Vienna, Austria

Correspondence: marco.hein@maxperutzlabs.ac.at (M.Y.H.), joe@derisilab.ucsf.edu (J.L.D.), weissman@wi.mit.edu (J.S.W.)

This Supplementary Information file contains:

Supplementary Figures 1–5

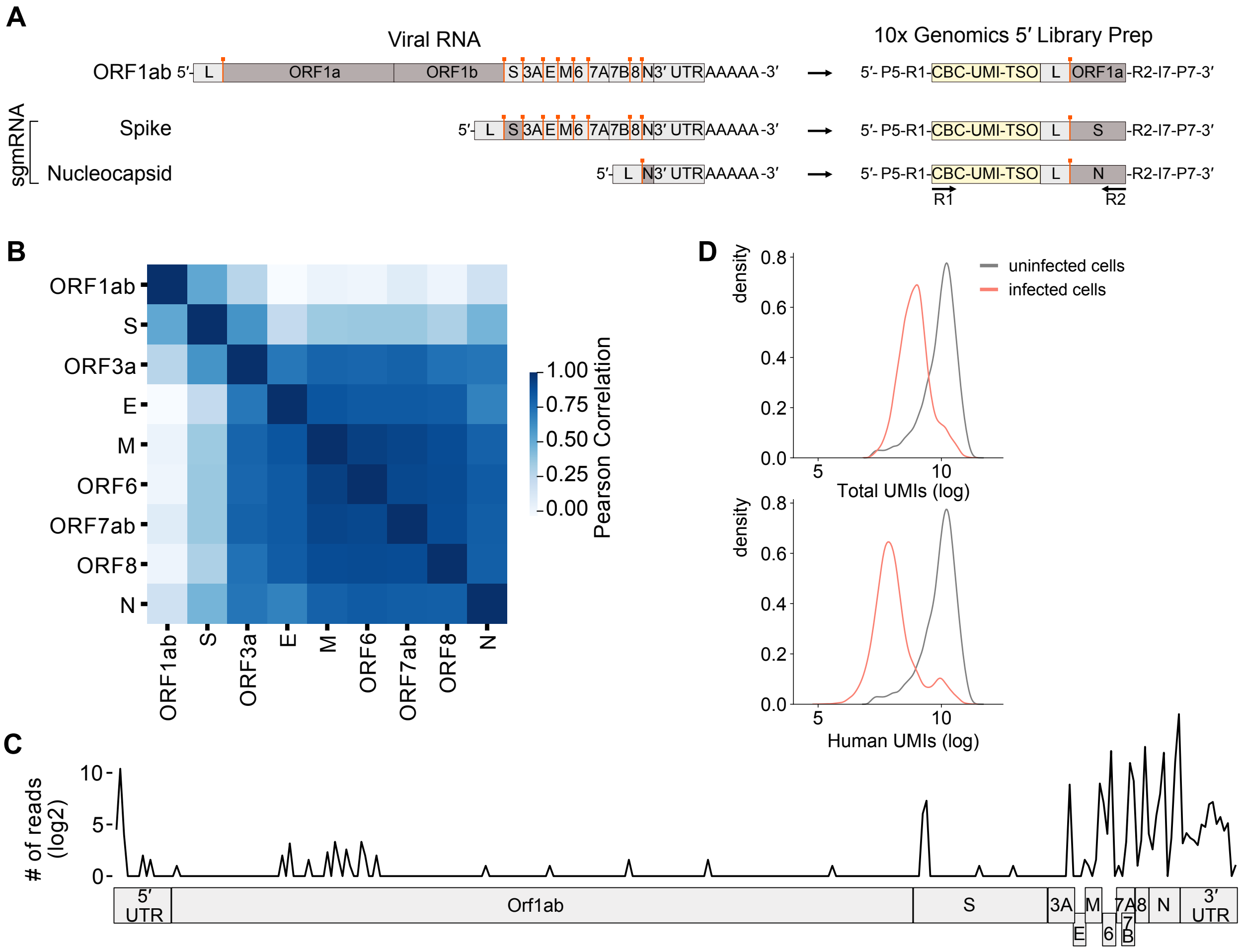

Supplementary Figure 1. Characterization of viral transcripts.

(A) Schematic representing the structure of SARS-CoV-2 viral RNA, and the expected sequence that captures the lead-body junction (represented with orange bar) using the 10x Genomics 5' workflow.
(B) Pearson correlation matrix of all viral RNA transcripts.
(C) Viral reads from infected cells were extracted and junction sites were mapped to the viral genome.
(D) Distribution of total cellular UMIs and UMIs assigned to human genes in infected and uninfected cells, respectively.

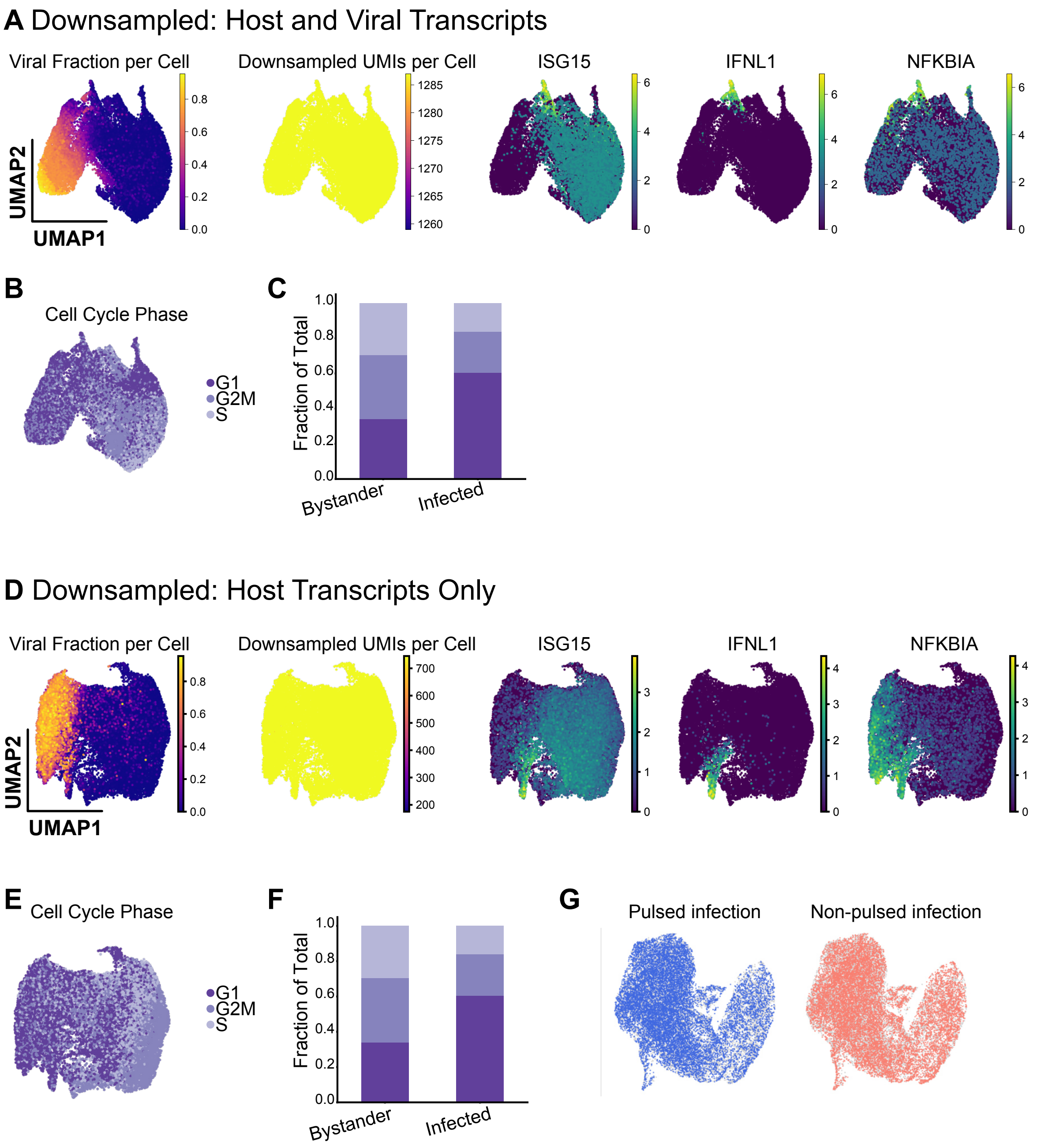

Supplementary Figure 2. Host gene expression and downsampling show similar transcriptional phenotypes.

(A–C) To confirm that transcriptional differences are not exclusively due to host shutoff, we downsampled host and viral transcripts and confirmed similar transcriptional patterns (A), and cell cycle phase patterns (B, C).
(D–F) We removed viral transcripts and performed the same analysis to confirm observed phenotypes are not an artifact of including viral reads in analyses.
(G) We confirmed that our two experimental conditions (pulsed and non-pulsed infection, respectively; see methods) contributed equally to the different observed cellular states.

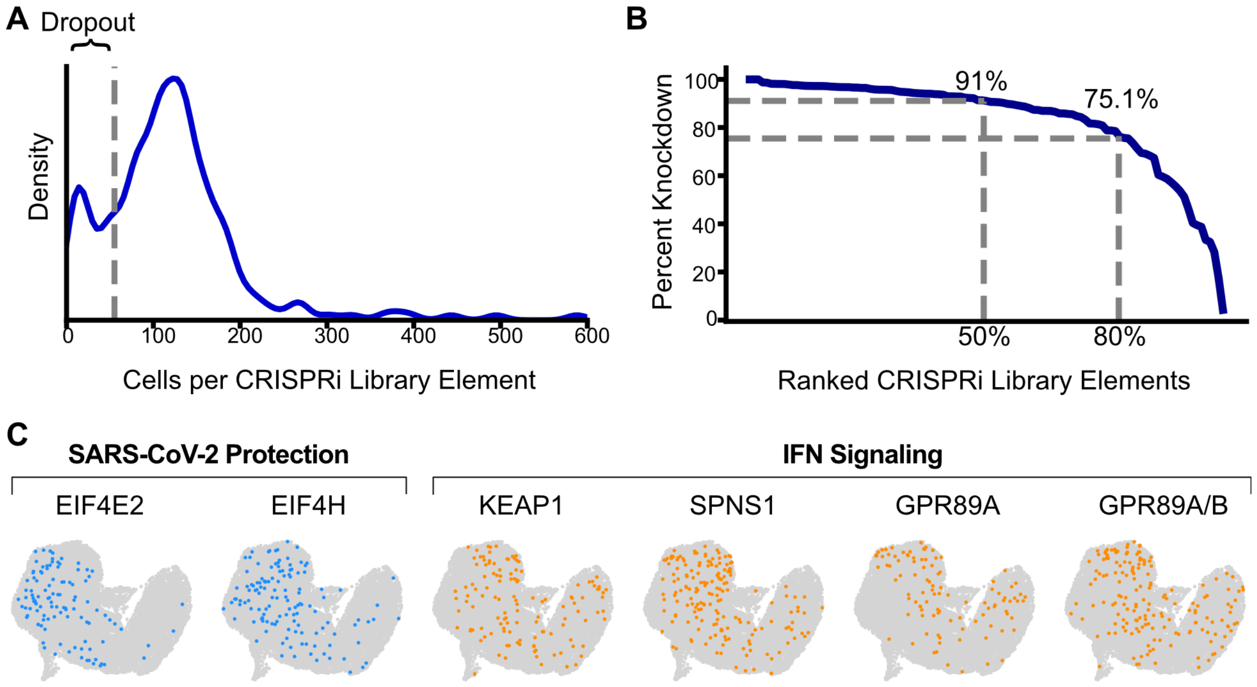

Supplementary Figure 3. CRISPRi library element representation and knockdown efficiency.

(A) We evaluated the distribution of captured cells for our 239 library elements and plotted the kernel density estimate.
(B) Knockdown percentage for each element was calculated relative to non-targeting controls and ranked by percent knockdown.
(C) In UMAP space, we highlighted the positions of cells containing selected library elements targeting factors that altered SARS-CoV-2 infection (blue) and interferon signaling (orange).

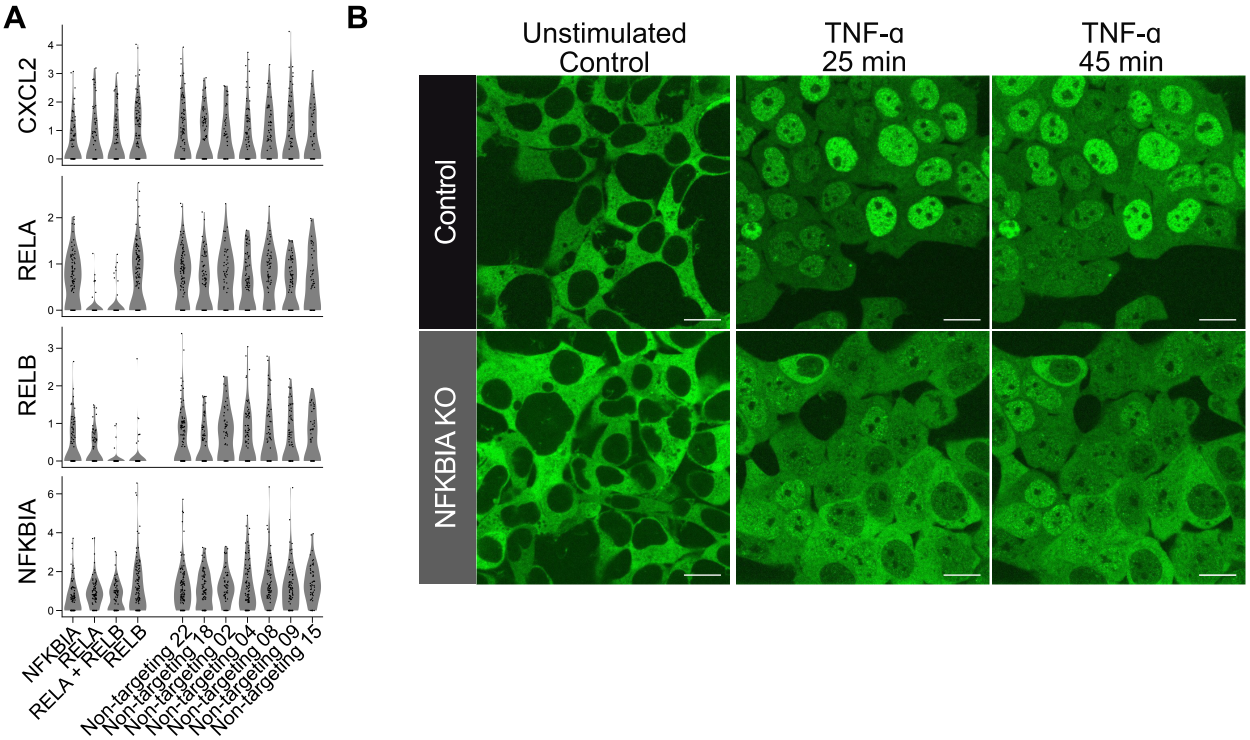

Supplementary Figure 4. Effect of NFKBIA perturbation on transcription and p65/RELA localization.

(A) To evaluate if NFKBIA knockdown transcriptionally alters NF-κB signaling in bystander cells, we looked at gene expression (log1p) of NF-κB target genes (CXCL2) as well as the pathway members themselves, which are on a negative feedback loop (RELA/B, NFKBIA) in cells with guides targeting NFKBIA, as well as RELA, RELB, both RELA and RELB, and non-targeting controls.

(B) To further investigate the effect of NFKBIA perturbation on p65/RELA localization, we utilized split mNeonGreen (mNG)-tagged RELA cells, generated polyclonal CRISPR NFKBIA knockout cells, and monitored p65/RELA localization at baseline. Additionally, we stimulated genetically unperturbed and NFKBIA KO cells with TNF-α and performed live-cell imaging at 25 and 45 minutes after stimulation (Scale = 20 μm). Experiment was repeated twice independently with similar results.

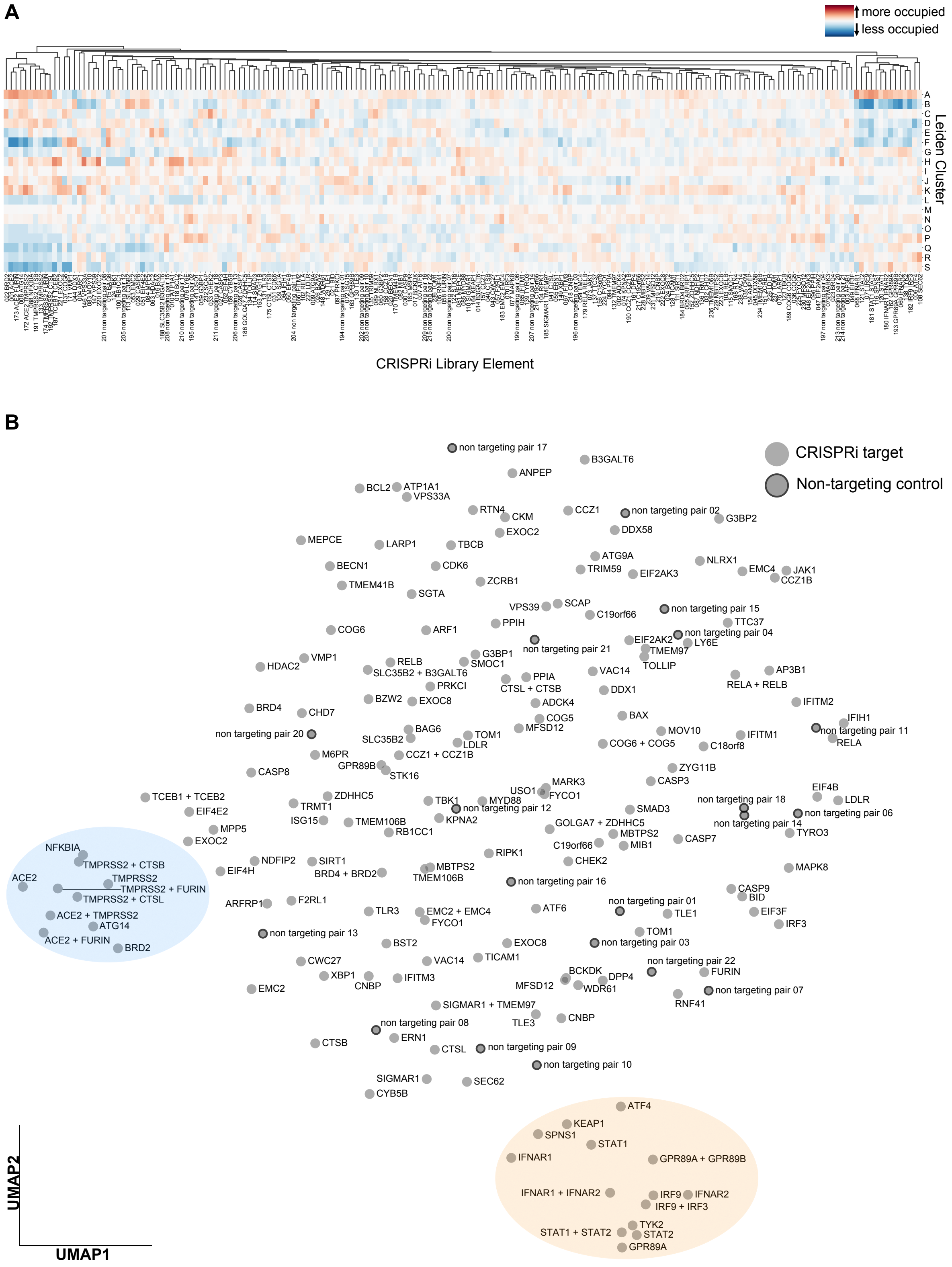

Supplementary Figure 5. Localization of CRISPRi library elements in Leiden clusters.

(A–B) Expanded version of (A) heatmap and (B) UMAP from Fig. 4 C, D, respectively, with all CRISPRi elements labeled.
