# Supplementary material for: Systematic functional interrogation of SARS-CoV-2 host factors using Perturb-seq
Source: Nat Commun. 2023 Oct 6;14:6245. doi: 10.1038/s41467-023-41788-4 (PMC10558542; doi:10.1038/s41467-023-41788-4)
Supplement: Supplementary file 3 — Description of Additional Supplementary Files Document [file 41467_2023_41788_MOESM3_ESM.pdf]

Description of Additional Supplementary File

Supplementary Data 1- Description: Perturb-seq library and host factor metadata.

Supplementary Data 2 - Description: Single-cell metadata.
