# Supplementary material for: Systematic functional interrogation of SARS-CoV-2 host factors using Perturb-seq
Source: Nat Commun. 2023 Oct 6;14:6245. doi: 10.1038/s41467-023-41788-4 (PMC10558542; doi:10.1038/s41467-023-41788-4)
Supplement: Supplementary file 6 — Reporting Summary [file 41467_2023_41788_MOESM6_ESM.pdf]

nature portfolio

Corresponding author(s): Marco Y. Hein
Joseph L. DeRisi
Jonathan S. Weissman

Last updated by author(s): Jul 28, 2023

Reporting Summary

Nature Portfolio wishes to improve the reproducibility of the work that we publish. This form provides structure for consistency and transparency in reporting. For further information on Nature Portfolio policies, see our Editorial Policies and the Editorial Policy Checklist.

Statistics

For all statistical analyses, confirm that the following items are present in the figure legend, table legend, main text, or Methods section.

n/a	Confirmed	
☐	☒	The exact sample size (*n*) for each experimental group/condition, given as a discrete number and unit of measurement
☐	☒	A statement on whether measurements were taken from distinct samples or whether the same sample was measured repeatedly
☐	☒	The statistical test(s) used AND whether they are one- or two-sided<br>*Only common tests should be described solely by name; describe more complex techniques in the Methods section.*
☒	☐	A description of all covariates tested
☒	☐	A description of any assumptions or corrections, such as tests of normality and adjustment for multiple comparisons
☐	☒	A full description of the statistical parameters including central tendency (e.g. means) or other basic estimates (e.g. regression coefficient) AND variation (e.g. standard deviation) or associated estimates of uncertainty (e.g. confidence intervals)
☐	☒	For null hypothesis testing, the test statistic (e.g. *F*, *t*, *r*) with confidence intervals, effect sizes, degrees of freedom and *P* value noted<br>*Give P values as exact values whenever suitable.*
☒	☐	For Bayesian analysis, information on the choice of priors and Markov chain Monte Carlo settings
☒	☐	For hierarchical and complex designs, identification of the appropriate level for tests and full reporting of outcomes
☐	☒	Estimates of effect sizes (e.g. Cohen's *d*, Pearson's *r*), indicating how they were calculated

Our web collection on statistics for biologists contains articles on many of the points above.

Software and code

Policy information about availability of computer code

Data collection	Single-cell data preprocessing: Cellranger v.3.1.0 (10x Genomics)<br>sgRNA calling: https://github.com/josephreplogle/guide_calling
Data analysis	Single-cell data analysis: ScanPy v.1.7.2 (Wolf et al, 2018), Seurat 4.1.0 (Hao et al. 2021).

For manuscripts utilizing custom algorithms or software that are central to the research but not yet described in published literature, software must be made available to editors and reviewers. We strongly encourage code deposition in a community repository (e.g. GitHub). See the Nature Portfolio guidelines for submitting code & software for further information.

Data

Policy information about availability of data

All manuscripts must include a data availability statement. This statement should provide the following information, where applicable:

- Accession codes, unique identifiers, or web links for publicly available datasets
- A description of any restrictions on data availability
- For clinical datasets or third party data, please ensure that the statement adheres to our policy

Raw and preprocessed data are available on GEO (accession GSE208240). Reference genomes utilized for this study include: hg38 and NC_045512.2.

Research involving human participants, their data, or biological material

Policy information about studies with human participants or human data. See also policy information about sex, gender (identity/presentation), and sexual orientation and race, ethnicity and racism.

Reporting on sex and gender	n/a
Reporting on race, ethnicity, or other socially relevant groupings	n/a
Population characteristics	n/a
Recruitment	n/a
Ethics oversight	n/a

Note that full information on the approval of the study protocol must also be provided in the manuscript.

Field-specific reporting

Please select the one below that is the best fit for your research. If you are not sure, read the appropriate sections before making your selection.

☒ Life sciences ☐ Behavioural & social sciences ☐ Ecological, evolutionary & environmental sciences

For a reference copy of the document with all sections, see nature.com/documents/nr-reporting-summary-flat.pdf

Life sciences study design

All studies must disclose on these points even when the disclosure is negative.

Sample size	The Perturb-seq experiment was conducted at ~138x coverage (i.e. typically 138 cells per library element per time point). This number was based on previous studies following a similar Perturb-seq workflow (Adamson et al., 2016; Replogle et al., 2022).
Data exclusions	Library elements represented by <50 cells were excluded from downstream analysis.
Replication	The Perturb-seq experiment was performed once, with two slightly different infection conditions (removal of the inoculum after 1h vs inoculum left for the entire 24h infection period). For downstream analyses, cells from both experimental conditions were combined. Since each cell with a given sgRNA library element constitutes one internal replicate measurement in a fully internally controlled experiment, we performed no replication of the overall experiment. Follow-up experiments involving microscopy were performed in two biological replicates.
Randomization	Randomization was not relevant to this study since our Perturb-seq library simultaneously tested the effect of perturbation of 183 host factors on SARS-CoV-2 infection.
Blinding	Blinding was not relevant to the Perturb-seq experiment in our study since all elements were tested simultaneously in the same cellular population. For our TCID50 orthogonal validation, the experimentalist was blinded to the gene-targeting condition.

Reporting for specific materials, systems and methods

We require information from authors about some types of materials, experimental systems and methods used in many studies. Here, indicate whether each material, system or method listed is relevant to your study. If you are not sure if a list item applies to your research, read the appropriate section before selecting a response.

Materials & experimental systems

n/a	Involved in the study	
☐	☒	Antibodies
☐	☒	Eukaryotic cell lines
☒	☐	Palaeontology and archaeology
☒	☐	Animals and other organisms
☒	☐	Clinical data
☒	☐	Dual use research of concern
☒	☐	Plants

Methods

n/a	Involved in the study	
☒	☐	ChIP-seq
☒	☐	Flow cytometry
☒	☐	MRI-based neuroimaging

Antibodies

Antibodies used	SARS-CoV/SARS-CoV-2 Nucleocapsid Antibody, Rabbit MAb (Sino Biological, Catalog: 40143-R001); Goat anti-Rabbit IgG (H+L) Highly Cross-Adsorbed Secondary Antibody, Alexa FluorTM 488 (Thermo Fisher Scientific, Catalog: A-11034).
Validation	The manufacturer has validated the primary antibody for Western blot, ELISA, Immunohistochemistry and Immunocytochemistry/ Immunofluorescence applications using recombinant N protein from SARS-CoV. It is cross-reactive with N proteins from all SARS-CoV-2 variants tested. The applications have not been validated with live virus. We performed no further validation experiments beyond those provided by the manufacturer.

Eukaryotic cell lines

Policy information about cell lines and Sex and Gender in Research

Cell line source(s)	Calu-3 and HEK293T cells were obtained from the UCSF Cell and Genome Core.<br>Huh7.5.1 cells overexpressing ACE2 and TMPRSS2 were obtained from Andreas Puschnik (see Wang et al, 2021).<br>HEK293T cells expressing endogenously tagged RELA were obtained from Manuel Leonetti (see Cho et al., 2022).
Authentication	Cells were not authenticated for this study.
Mycoplasma contamination	All cell lines were tested on a regular basis to confirm absence of mycoplasma contamination.
Commonly misidentified lines (See ICLAC register)	No commonly misidentified cell lines were used.
